# Mantle heterogeneity caused by trapped water in the Southwest Basin of the South China Sea

**DOI:** 10.1038/s41467-023-38385-w

**Published:** 2023-05-11

**Authors:** Jinyu Tian, Zhitu Ma, Jian Lin, Min Xu, Xun Yu, Ba Manh Le, Xubo Zhang, Fan Zhang, Laiyin Guo

**Affiliations:** 1 Department of Ocean Science and Engineering, Southern University of Science and Technology, Shenzhen, 518055 China; 2 State Key Laboratory of Marine Geology, School of Ocean and Earth Science, Tongji University, Shanghai, 200092 China; 3 Key Laboratory of Ocean and Marginal Sea Geology, South China Sea Institute of Oceanology, Innovation Academy of South China Sea Ecology and Environmental Engineering, Chinese Academy of Sciences, Guangzhou, 511458 China

**Keywords:** Seismology, Geophysics

## Abstract

Water is the most common volatile component inside the Earth. A substantial amount of water can be carried down to the interior of the Earth by subducting plates. However, how the subducted water evolves after the subducting slab breaks off remains poorly understood. Here we use the data from a passive seismic experiment using ocean bottom seismometers (OBSs) together with the land stations to determine the high-resolution, three-dimensional seismic structure of the Southwest Sub-basin (SWSB) of the South China Sea (SCS). At depths below 40 km, the mantle shear velocity (Vsv) beneath the northern side of the SWSB is similar to that of the conventional oceanic pyrolite mantle, but roughly 3% shear-velocity reduction is found beneath the southern side of the SWSB. Results of thermal dynamic modeling reveal that the observed shear-velocity reduction could be explained by the presence of 150–300 ppm of water and 5–10% of lower continental crust. The inferred high-water content at the southern side of the SWSB is consistent with a model in which the Proto-SCS plate subducted southward prior to and during the formation of the SCS basin, releasing water into the upper mantle of the SWSB.

## Abstract (web summary)

This study suggests that the observed shear-velocity reduction beneath the southern side of the Southwest Sub-basin (SWSB) of the South China Sea (SCS) may be due to the presence of 150–300 ppm of water and 5–10% of lower continental crust.

## Introduction

The distribution and circulation of water have important effects on the evolution of Earth^1^, and subduction zones are crucial sections where water could be transported into deep Earth. This process has been investigated extensively around the Pacific Ocean using seismological and magnetotelluric observations^2–6^. During plate subduction, dehydration of the hydrated sediment, oceanic crust, and the uppermost mantle would release fluids into the overlying mantle wedge to trigger arc melting^7^. The hydrated mantle layer on top of the subducting plate also provides a pathway for water into the deep mantle^8^. However, the distribution of water in the overlying mantle and the evolution of water after the breaking off of the subducting slab are still poorly investigated. The South China Sea (SCS), located in the western Pacific Ocean, is an ideal place to address these questions where the Proto-SCS (PSCS) plate has been subducted and broke off beneath the northern Borneo and Nansha Block^9^.

**Fig. 1:**
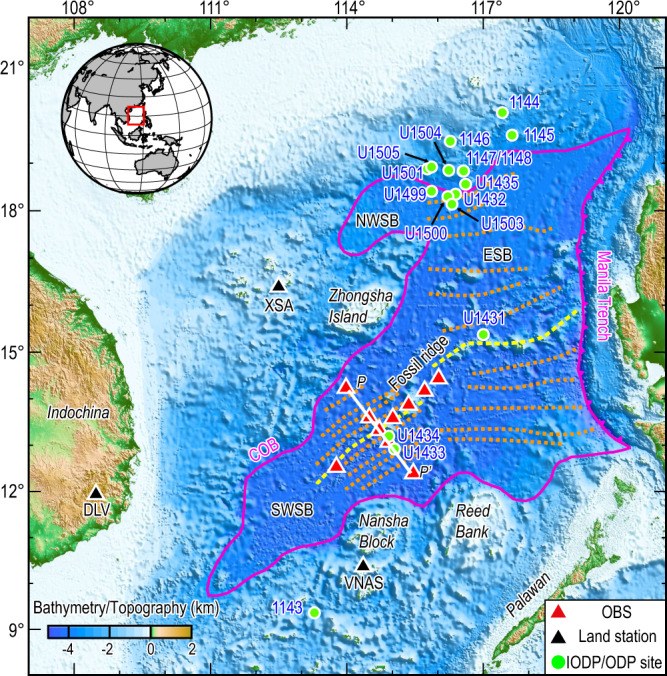
Bathymetry map with major tectonic features shown of the study region. The red and black triangles indicate the locations of ocean bottom seismometers (OBSs) and land stations used in this study, respectively. The white line indicates the position of the profile P-P’. The study region is marked with a red frame in the inset. Green dots mark the locations of the International Ocean Discovery Program (IODP)/Ocean Drilling Program (ODP) Expedition sites. The yellow and orange dashed lines represent the fossil spreading ridge and the magnetic lineations^10^. The solid pink line represents the continent-ocean boundary (COB) of the South China Sea (SCS) basin, and the solid pink line with triangles indicates the Manila trench. NWSB Northwest Sub-basin, ESB East Sub-basin, SWSB Southwest Sub-basin.

The seafloor spreading of the SCS began at ~32 Ma in the East Sub-basin, expanded westward subsequently, and ceased at ~16 Ma^10^. The Southwest Sub-basin (SWSB) of the SCS has an opening history of ~23 to 16 Ma and a V-shaped geometry. The relatively short opening history of the SWSB facilitates it to retain important information about continental rifting and basin evolution. To infer upper mantle heterogeneity characteristics of the SWSB, previous studies mainly relied on geochemical observations^11–14^, geodynamical simulations^15,16^, seafloor morphology^15^, and crustal structure^17,18^. The International Ocean Discovery Program (IODP) Expedition349 collected samples of the igneous oceanic crust around the SWSB fossil ridges (Sites U1433/U1434, Fig. 1). Numerous analyses of these basalt samples indicated complex mantle source compositions beneath the SWSB^13,16,19,20^. The comparatively lower ^206^Pb/^204^Pb ratio in these basalt samples indicated that ~5% of the lower continental crust (LCC) was trapped in the underlying mantle during the continental rifting and breaking process^11,13^. On the other hand, the presence of water could also cause mantle heterogeneity. Prior geochemical studies have proposed that the water content of the mantle beneath the SCS basin might be contributed by the Hainan plume^14^, while the seismic receiver function analysis shows that the water could also be contributed by the PSCS slab dehydration at the Nansha Block^9^. Several key questions remain unanswered: (1) Geochemical heterogeneity of the mantle was investigated based on restricted basalt samples from IODP sites U1433/U1434; there is still a lack of studies to quantify the amount of LCC throughout the entire SWSB. (2) The role of water in generating mantle seismic velocity anomalies has not been quantified, and the source of the water is controversial. (3) The relationship between the subduction direction of the PSCS and mantle heterogeneity remains unclear. The majority of the research assumed that the PSCS slab was subducted southward, while other studies suggested that a slab segment has subducted northward^21,22^.

To better investigate the upper mantle structure of the SWSB, we conducted a broadband ocean bottom seismometers (OBSs) experiment near the fossil ridge in the SWSB onboard R/V Shiyan 3 of the South China Sea Institute of Oceanography from June to December 2017 (Fig. 1). In this study, we first determined a three-dimensional (3-D) isotropic shear-velocity model of the upper mantle in the SWSB via surface wave tomography, using seismic data recorded by the OBSs and land stations (Fig. 1). Then, we performed a detailed analysis of the trade-off effects on the shear-velocity structure by a combination of mantle temperature, major element composition, and water content. Finally, a new model of the SWSB evolution was proposed to explain the observed anomalies in terms of the SCS opening and PSCS slab subduction.

## Results and discussion

### Seismic tomography

**Fig. 2:**
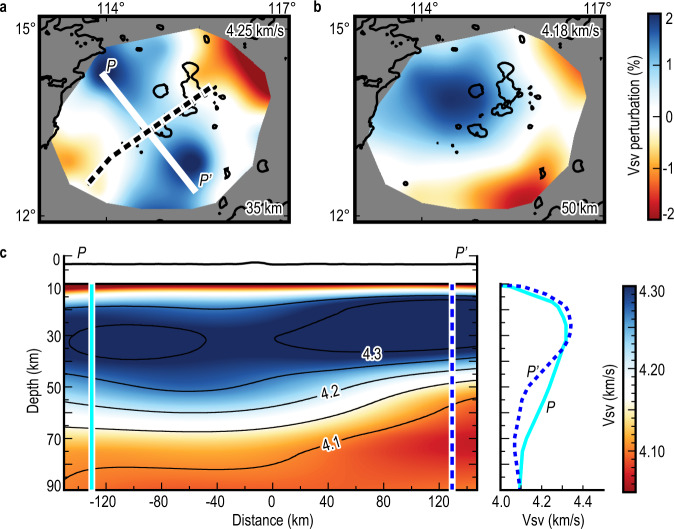
Mantle shear-velocity models of the Southwest Sub-basin of the South China Sea. The azimuthally averaged vertically polarized S-waves velocity (Vsv) perturbations at depths of (**a**) 35, (**b**) 50 km are calculated by the average phase velocity, shown in the panels’ top right. Thick white line shows the location of the cross-section in (**c**). Black dashed line represents the fossil spreading ridge. **c** Cross-section of Vsv along profile P-P’. Vertical cyan solid and blue dashed lines mark the locations of the one-dimensional Vsv structure shown on the right of (**c**).

**Fig. 3:**
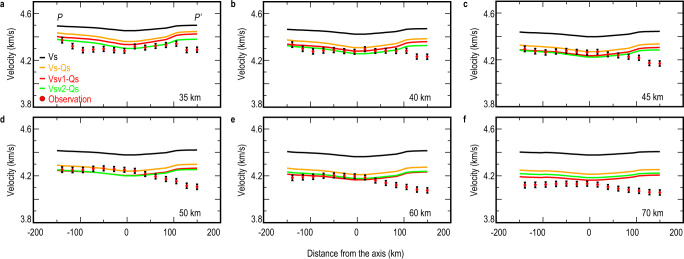
Comparison of predicted and observed vertically polarized S-waves velocity (Vsv) models. These models were along the cross-section P-P’ at depths of (**a**) 35, (**b**) 40, (**c**) 45, (**d**) 50, (**e**) 60, and (**f**) 70 km. The red dots with vertical black lines are the observed Vsv and estimated uncertainty bars. Black line: initially predicted shear velocity (Vs) calculated from the HeFESTo program^30–32^; Gold line: the predicted Vs after shear attenuation (Qs) correction; Green line: the predicted Vsv after Qs correction and radial anisotropy correction^35^ (SEMum2 model); Red line: the predicted Vsv after Qs correction and radial anisotropy correction^34^ (S362ANI + W model).

The azimuthally averaged velocity model of vertically polarized S-waves velocity (Vsv) was first determined. The results show that the shear-velocity models are approximately the same (~4.30 km s^−1^) at the depth of ~35 km on both sides of the fossil ridge (Fig. 2a, c and Supplementary Fig. 6a). However, as depth increases, the Vsv difference between the conjugated sides of the fossil ridge becomes larger, with the most significant Vsv difference (~3%) occurring at the depth of ~50 km (Fig. 2b, c and Supplementary Fig. 6b–d). A substantial shear-velocity reduction area with an N-S width of 100–150 km was identified at 40–70 km depth beneath the southern end of the SWSB study region (Figs. 2c and 3). In this shear-velocity reduction area, the slowest Vsv for the southern side is 4.07 km s^−1^ at 70 km deep, while the northern side is 4.13 km s^−1^ at the same depth (Fig. 2c). The lateral resolution of the shear-velocity model is 1° (Supplementary Fig. 3); the sensitivity kernels show that the Vsv structure at depths ranging from 30 to 80 km could be well resolved (Supplementary Fig. 6e). The identified Vsv uncertainties are estimated to be 1% from the inversion, which is significantly smaller than the velocity anomalies (Fig. 3). This shear-velocity reduction on the southern side of the fossil ridge was not found in previous seismic models based purely on land stations^23–25^, emphasizing the importance of local OBS stations in increasing tomographic resolution of the SWSB.

### Predicting Vsv structure from the standard oceanic cooling model

Previous studies proposed several oceanic lithospheric thermal models, such as the half-space cooling model^26^, plate cooling model^27,28^, and the CHABLIS model^29^. These models provide a quantitative relationship between the plate age and 1-D depth-related mantle temperature. The mantle temperature structures estimated from these models are very similar for the young plate (<50 Ma) in the SWSB, and we employ the half-space cooling model^26^ with a 1600 K potential mantle temperature (Tp) of infinite depth. To connect mantle temperature to seismic velocity, we used the HeFESTo program^30–32^ (see “Methods”) to estimate the shear-velocity model (Fig. 3, black lines) along the section P-P’ across the fossil ridge (Fig. 2a). The extended Burgers model^33^ (See Methods) was used to correct for the effect of shear attenuation (Qs) on the seismic wave (Fig. 3, gold lines). Finally, two existing global seismic models, S362ANI + W^34^ (Fig. 3, red lines) and SEMum2^35^ (Fig. 3, green lines), were used to correct for the radial anisotropy. The comparison shows that the difference (<0.03 km s^−1^) between the Vsv corrected for these two anisotropic models can be ignored.

The first-order observation is that the overall seismic structure of the northern SWSB can indeed be well-fitted by this standard oceanic cooling model (Fig. 3). The predicted Vsv models are well within the uncertainty (0.05 km s^−1^) for the northern SWSB below 40 km depth. This demonstrates that, despite its relatively short opening history and complex geological surroundings, the evolution of the northern SWSB follows the same evolution trend as typical ocean basins. However, the southern end of the SWSB study region deviates significantly from this simple model. Potential causes for this discrepancy are discussed below.

### Factors controlling shear-velocity reduction

The noticeable shear-velocity reduction can be caused by a variety of variables, including mantle temperature, composition, water content, and melting^33,36^, which will be discussed in detail.

The seismic velocity decreases as mantle temperature rises. We quantified the predicted Vsv by varying the mantle temperature along the cross-section P-P’ (Supplementary Fig. 7) and fitting the observed anomalies (Supplementary Fig. 10a). At depths of 35–70 km beneath the southern SWSB, an increase in mantle temperature of up to 300 K is required, which could have caused significant melting of the entire mantle. However, seismic profiles^18^ and gravity inversion^15^ revealed that the crust in the northern half of the SWSB is thicker than that in the southern half. The thicker crust implies enhanced magma activity during the basin opening^15^. Furthermore, the heat flow on the northern side of the SWSB is higher than that on the south side^37^. The above observations are inconsistent with models of the southern SWSB having a higher mantle temperature or greater degree of mantle melting. Thus, we infer that increased mantle temperature and/or partial melts caused by increased temperature are not the major factors contributing to the shear-velocity reduction in the southern SWSB.

Seismic velocity is also sensitive to mantle composition^30,31,38,39^. It is widely assumed that the normal oceanic mantle is composed of a fertile peridotite (pyrolite) that melts to form the mid-ocean-ridge basalt (MORB)^40^. Here, we investigated how mantle composition can affect seismic velocities using the HeFESTo program^30–32^ (see “Methods” and Supplementary Fig. 8). Recycling oceanic crust in subduction zones can account for the chemical composition anomalies of enriched mantle sources^41^. We quantified the amount of MORB mixed into the pyrolite mantle that is required to explain the observed Vsv model along the cross-section P-P’ (Supplementary Figs. 9 and 10b). A greater amount of MORB is required as the depth increases. Due to the phase transition from quartz to coesite at ~70 km depth^39^, the basalt will turn into eclogite, which increases the Vsv, in contrast to our observed velocity reduction below 70 km depth (Supplementary Fig. 9f). Therefore, we infer that MORB has no significant impact on the shear-velocity reduction beneath the southern end of the SWSB.

**Fig. 4:**
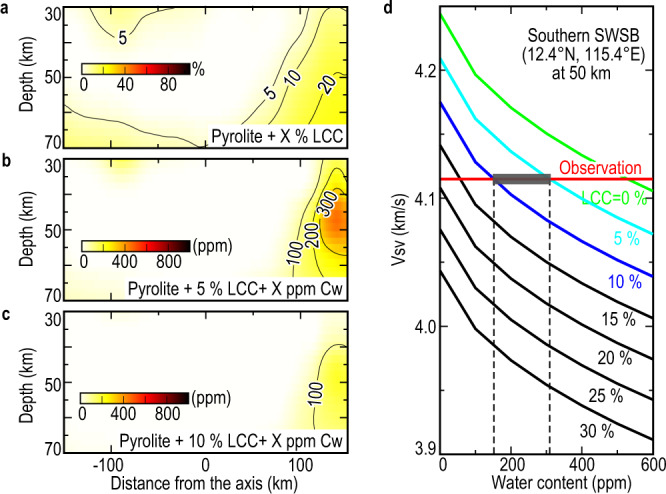
Best fitting models of mantle composition variations along the cross-section P-P’. Fitting the observed vertically polarized S-waves velocity (Vsv) structure by changing different mantle conditions: **a** adding the lower continental crust (LCC) into the pyrolite mantle; **b** adding the structurally bound water (Cw) and 5% of LCC into the pyrolite mantle; **c** same as (**b**), but with 10% LCC. **d** Predicted Vsv models calculated by adding various amounts of water and LCC into the pyrolite mantle at 50 km depth of an example location in the Southern SWSB (12.4°N, 115.4°E). The red horizontal line is the observed Vsv. The gray shaded area predicts the amount of water and LCC.

We then tested a model of mantle mixed with LCC (Fig. 4a and Supplementary Fig. 11a–f). The bulk composition of LCC is taken from the global average model^42^ (Supplementary Table 1). On the northern side of the SWSB, a model with ~5% LCC is consistent with the observed Vsv above 40 km depth. This finding is compatible with previous geochemical studies^11,13^, which were based on the Sr-Nd-Pb-Hf isotopic ratios of basalt samples from sites U1433 and U1434 (Fig. 1). However, the shear-velocity reduction beneath the southern end of the SWSB could only be fitted with a mantle model with 20% LCC. Such an amount of LCC beneath the southern end of the SWSB estimated in this study is much larger than that calculated from these studies (~5%), suggesting that there are other factors contributing to the shear-velocity reduction. We also constructed the LCC using the bulk compositions of the South China Block samples^43^ (Leizhou and Qilin), and the fitting models were similar to the global average model (Supplementary Fig. 11g, h).

Structurally bound water could affect seismic velocity via anelastic behavior^44^. The empirical relationship between water content and grain-boundary viscosity^5,44^ can be used, together with the extended Burgers model^33^, to predict the Vsv anomaly. Generally, the water concentration at mid-ocean ridges rarely exceeds 100–500 ppm^45^. The water content in the mantle source of the southeastern Asian basalt province (SABP) was estimated to range between 84 and 360 ppm according to the measurement of the Cenozoic Hainan basalts^14^, while the samples of seamounts in the SCS revealed a similar result^46^. However, we found that if no LCC is involved, more than 500 ppm of water in the mantle is required to explain the observed Vsv anomaly at 50 km depth beneath the southern SWSB (Fig. 4d, green line). When the mantle is mixed with up to 5% LCC, ~300 ppm water is still required to explain the observed Vsv beneath the southern end of the SWSB (Fig. 4b). If the mantle is mixed with a greater amount of LCC (e.g., 10%), a decreased water content of ~150 ppm is required in the mantle (Fig. 4c). Since neither too much water nor LCC in the mantle makes sense, we infer that the amounts of LCC and water should be ~5–10% and ~150–300 ppm, respectively (Fig. 4d, gray shaded area).

We also investigated the possibility of pondering melts on the southern side of the SWSB due to the water-enhanced mantle melting. According to ref. ^47^ (see “Methods”), we first calculated the effect of water content in nominally anhydrous minerals on the solidus of pyrolite mantle (Supplementary Fig. 12a). Then, the water concentration necessary to stabilize a melt along the geotherm of P’ (12.4°N, 115.4°E) and the amount of water generated in near-solidus partial melts in the pyrolite mantle were also estimated in this study (cyan and blue lines, Supplementary Fig. 12b). Hydrous melting will occur if the near-solidus partial melt has more water than is required for partial melting at that depth; that is, the blue line has to be higher than the red line (Supplementary Fig. 12b). The analysis reveals that the shear-velocity reduction estimated in this study is not likely to have been caused by significant additional water-enhanced mantle melting.

Regarding the possibility of other compositions such as MORB or LCC in the mantle causing additional melting, previous petrological experiments have suggested that the silicon-rich eclogite-derived melts could react with surrounding peridotite and refertilize it^48,49^; and thus, it is difficult for melts derived from relatively low-degree partial melting of MORB or LCC to exist in the upper mantle. To conclude, we believe that the shear-velocity reduction at the southern end of the SWSB is most likely caused by a combination of the presence of water and LCC and less likely by rising mantle temperature or the presence of melts.

### Mantle evolution of the SWSB

The above quantitative analysis indicates that water and LCC could both contribute to the mantle evolution of the SWSB. For the source of water, previous geochemical studies suggested that the Hainan plume might have caused the opening of the SCS basin^11–13^ and carried a significant amount of water to the mantle beneath the SCS^14^. However, if this was the case, the Vsv beneath the basin on both sides of the fossil ridge should have been similar. We infer that the water could be released from the PSCS slab. There are three main lines of evidence to support this inference: (1) shear wave splitting models^50,51^ show the direction of the deep mantle flow beneath the Sunda plate is NW-SE; (2) the absolute plate motion of the Sunda plate is observed to be southeastward with a speed of ~32 mm a^−1 ^^52^ (NNR-MORVEL56 model); (3) geophysical and geological studies suggest the existence of post-subducted PSCS slab segments beneath the northern Borneo^9,23,24^. As a result, we infer that the PSCS slab segments broke and sank into the deep mantle after the SCS spreading stopped (~16 Ma). The Sunda plate has moved about 500 km southeastward since then, making the falling PSCS slab segments currently situate beneath the SWSB, which is consistent with observations^9,23,24^. In particular, receiver function analysis of the mantle transition zone beneath the Borneo and the southern SCS revealed that a significant amount of water might have leaked from the PSCS slab^9^. Therefore, we suggest that the water, as the main control factor contributing to the shear-velocity reduction beneath the southern SWSB, might have been released from the falling PSCS slab segments.

**Fig. 5:**
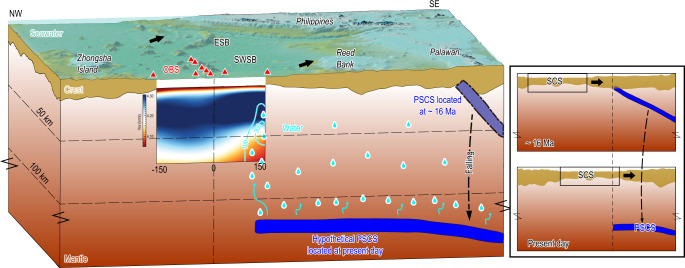
A conceptual cartoon for the evolution of the Southwest Sub-basin of the South China Sea. The color scale of the Vsv model along profile P-P’ is the same as in Fig. 2c. The contour lines of water content in the Vsv model are from Fig. 4b. The red triangles indicate the location of ocean bottom seismometers (OBS) stations. Cyan drops denote releasing of dehydrated water. Cyan curves with arrows indicate the dehydration of the Proto South China Sea (PSCS) slab. Black dashed line with an arrow shows the sinking direction of the PSCS slab. After the South China Sea (SCS) spreading stopped at ~16 Ma (right top panel), the PSCS slab segments sank into the deep mantle; since the Sunda plate has moved about 500 km southeast, the falling PSCS slab segments are now located beneath the SWSB (right bottom panel). The black arrow marks the direction of absolute plate motion. The morphology and evolution of the PSCS slab are referred to in ref. ^9^. ESB East Sub-basin, SWSB Southwest Sub-basin.

Based on the above analysis, we propose a new model of the upper mantle evolution beneath the SWSB (Fig. 5). When the SWSB began to spread, the underlying mantle was contaminated by the LCC due to crustal delamination^11,16^. The most important driving force for LCC delamination is negative buoyancy caused by the density difference between the LCC/lithosphere and ambient mantle, which induces gravitational instability leading to lithosphere removal^53–55^. The combined effects of the upwelling mantle force, increased thermal buoyancy, and/or removal of the dense components as the delaminated LCC sinks are hypothesized to keep the LCC materials in the upper mantle^56^. The delaminated LCC may spread widely in the upper mantle due to mantle convection and experience decompression melting when seafloor spreading occurs. As a result, geochemical studies can detect LCC signals in extensive basaltic lavas in the SCS^11,20,57^. As the contaminated mantle underwent decompression melting and progressively drifted away from the ridge axis, the deeper unmodified mantle column moved upward and occupied the fossil ridge mantle source region. This mechanism would cause the amount of the LCC in the mantle to decrease gradually. Since the SCS spreading stopped, a significant amount of water might have been released from the falling PSCS slab, causing a relatively high water content (~150–300 ppm) beneath the southern end of the SWSB (Fig. 5). Meanwhile, the comparable Vsv between the northern side of the SWSB and the normal oceanic plate weakens the argument of a northward subducted PSCS slab in the latest Cretaceous and the influence of Hainan plume on the SWSB evolution. Further local seismic experiments could provide additional constraints on the crucial water content of the entire SCS as well as the evolution process of the SCS.

## Methods

### Seismic data processing and inversion

Data for phase velocity tomography were obtained by ten OBSs and three land stations (Fig. 1). We selected seismograms of 91 earthquakes with surface-wave magnitudes (Ms) larger than 4.5 and epicentral distances between 20° and 150° that occurred during the OBS deployment period from the Incorporated Research Institutions for Seismology (IRIS) catalog (Supplementary Fig. 1). The raw seismogram of each event was cut from the original time of the earthquake to 7200 s afterward. Before phase velocity inversion, vertical seismograms were down-sampled to 5 Hz, while instrument responses were removed. The improved noise cross-correlation function methods were used to correct time errors in the OBS data^58^. High-quality seismograms were then windowed, tapered, and Fourier transformed to determine the amplitude and phase at each discrete period from 20 to 50 s.

We applied the two-plane-wave tomography (TPWT) method^59^ with two-dimensional (2-D) Fréchet kernels^60^ to determine azimuthally averaged phase velocities of the fundamental mode Rayleigh waves in the range of 20–50 s. Traditional Rayleigh wave tomography assumes the wave travels along the great circle path from source to receiver. The TPWT approach analyzes the incoming wavefield of the Rayleigh wave as the sum of two interfering plane waves to account for the non-great-circle propagating paths^59^. Each plane wave has its amplitude, initial phase, and direction, so each event requires the solution of six wavefield parameters. The scattering and focusing effects caused by the heterogeneities near their propagating paths can be approximately estimated from the 2D Fréchet kernels^60^. The TPWT method iterates in two steps. In the first step, the incoming two plane waves for each event are estimated using the initial phase velocity model. In the second step, a linearized damped iterative least-squares inversion is used to solve the wavefield parameters and phase velocities at each node.

We parametrized the velocity model using a grid of nodes with a grid spacing of 0.25°. We experimented with smoothing lengths ranging from 50 to 100 km and damping parameters of 0.10 to 0.30. An example of the 25 s inversion is shown in Supplementary Fig. 2. The phase velocity models are comparable near the fossil ridge of the SWSB. In this case, values of 80 km and 0.2 km s^−1^ for the smoothing length and velocity damping parameters were chosen, respectively. To determine the resolution of the phase velocity model, the resolution matrices were represented as 1° checkerboards, and the input model contains ±4% anomalies relative to the average velocity of 4.0 km s^−1^ (Supplementary Fig. 3a). The checkerboard analysis suggests a lateral resolution of the inversion on the order of 1° (Supplementary Fig. 3). It is worth noting that the lateral width of seismic velocity reduction beneath the southern end of the SWSB is about 100–150 km (Fig. 3), which is beyond the resolution of the inversion.

We also estimated the standard deviation of phase velocity from the model covariance matrix. The phase velocity uncertainty is around 0.035 km s^−1^ near the fossil ridge (Supplementary Fig. 4f). After these tests on the robustness of phase velocity maps, a linearized inversion^61^ was utilized to invert the azimuthally averaged Vsv at each node based on the phase velocity dispersion curve (Supplementary Fig. 4). The starting model of the inversion consists of four layers on top of a half-space: (1) water^62^; (2) sediments^15^; (3) crustal thickness^15^; and (4) upper mantle from the Moho to a depth of 200 km. The starting model of Vsv at each node is retrieved from CRUST1.0^63^ for water, sediment, and crust and IASP91^64^ for mantle. The resulting Vsv model fits very well the observation of Rayleigh wave dispersion curves (Supplementary Fig. 5a–b, red and blue solid curves), and the sensitivity kernel curves suggest that the reliable solution of Vsv is between 30 and 80 km (Supplementary Fig. 6e). To verify the robustness of the shear-velocity reduction, the Bayesian Monte Carlo method^65^ (MC) was also used to invert the Vsv models at the southern and northern side of the SWSB (Supplementary Fig. 5c). The starting model of the layer thicknesses and Vsv models in the MC method were comparable to those of the linearized method, but the thicknesses of water, sediment, and crust were allowed perturbations of ±1.0 km, ±0.5 km, and ±5.0 km, respectively; the sedimentary and crustal Vsv models were both allowed perturbations of ±1.0 km s^−1^; the upper mantle Vsv is parameterized by a *B*-spline. The difference in the Vsv models between the linearized method and MC method was around 0.5% between 40 and 70 km, which is significantly less than the observed shear-velocity reduction (3%). The results of the preceding tests demonstrate that calculated shear-velocity reduction beneath the southern end of SWSB is relatively robust.

### Thermodynamic models

The HeFESTo program^30–32^ and the extended Burgers model^33^ were used to predict the Vsv structure. The HeFESTo is built on fundamental thermodynamic relations that capture phase equilibria and physical properties. It can calculate the seismic velocity under various temperatures, pressures, and bulk compositions. The extended Burgers model^33^, constrained by experimental data on olivine samples, provides a link relating temperature and pressure to Qs.

The HeFESTo was used to estimate the Vsv models of different materials that vary with depth (Supplementary Fig. 8) by changing the bulk composition^16,39,42,43,66^ (Supplementary Table 1). The Mechanical Mixture (MM) disequilibrium end-member model was utilized to mix MORB/LCC into the pyrolite mantle (Fig. 4a–c and Supplementary Figs. 9–11). The elastic moduli of lithologic assemblages were calculated using the Voigt-Reuss-Hill averages of the constituent materials^39^. The relaxation times of the extended Burgers model were adjusted to estimate the effect of water using the empirical relationship between water content and grain-boundary viscosity based on the assumption that grain-boundary viscosity relies on water content^5,36,44^.

### Influence of water concentration in the melt on the dry solidus

The relationship between the water concentration and the freezing point depression relative to the dry solidus of the pyrolite mantle can be estimated by calculating the water concentration in near-solidus liquids^47^:

1 $${T}=\frac{{{T}}_{{{\mbox{peridotite}}}}^{{{\mbox{fusion}}}}}{\left(1-\left(\frac{{R}}{{M}\Delta {{S}}_{{{\mbox{peridotite}}}}^{{{\mbox{fusion}}}}}\right){{{{\mathrm{ln}}}}}\left(1-{{X}}_{{{{\mbox{OH}}}}^{-}}^{{{\mbox{melt}}}}\right)\right)}$$

where *T* is the temperatures of partial melting, $\Delta {S}_{{{{{{{\rm{peridotite}}}}}}}}^{{{{{{{\rm{fusion}}}}}}}}$ (0.4 J/K/g) is the entropy of fusion per unit mass, *M* (59 g/mole) is the number of grams in one mole of silicate, *R* is the gas constant, ${X}_{{{{{{{{\rm{OH}}}}}}}}^{-}}^{{{{{{{\rm{melt}}}}}}}}$ is the mole fraction of hydroxyl in the melt at the solidus. ${X}_{{{{{{{{\rm{OH}}}}}}}}^{-}}^{{{{{{{\rm{melt}}}}}}}}$ can be calculated by the following equation^67^:

2 $${{{X}}}_{{{{{{{\rm{OH}}}}}}}^{-}}^{{{{{{\rm{melt}}}}}}}=\frac{\frac{2\times {{{{{\rm{wt}}}}}}{.\%{{{{{\rm{H}}}}}}}_{2}{{{{{{\rm{O}}}}}}}_{{{{{{\rm{melt}}}}}}}}{18}}{\frac{2\times {{{{{\rm{wt}}}}}}{.\%{{{{{\rm{H}}}}}}}_{2}{{{{{{\rm{O}}}}}}}_{{{{{{\rm{melt}}}}}}}}{18}+\frac{100-{{{{{\rm{wt}}}}}}{.\%{{{{{\rm{H}}}}}}}_{2}{{{{{{\rm{O}}}}}}}_{{{{{{\rm{melt}}}}}}}}{{{M}}}}$$

${T}_{{{{{{{\rm{peridotite}}}}}}}}^{{{{{{{\rm{fusion}}}}}}}}$ represents the dry temperature and is a function of pressure (*P*). The formula for 1–10 GPa is as follows^68^:

3 $${{{T}}}_{{{\mbox{peridotite}}}}^{{{\mbox{fusion}}}}={-5.140{{P}}}^{2}+132.899{{P}}+1120.661$$

## Supplementary information

Supplementary Information

## Data Availability

The waveform data of OBSs and land stations are available upon request at the Key Laboratory of Ocean and Marginal Sea Geology (CAS, http://www.omg.scsio.ac.cn/), the Data Management Centre of China National Seismic Network at the Institute of Geophysics, China Earthquake Administration^69^ (SEISDMC, 10.11998/SeisDmc/SN, http://www.seisdmc.ac.cn), Broadband Array in Taiwan for Seismology^70^ (BATS, 10.7914/SN/TW, http://bats.earth.sinica.edu.tw), and IRIS Data Management Center (http://ds.iris.edu/ds/nodes/dmc/data/).

## Code availability

The map was made with Generic Mapping Tools (GMT) (https://www.generic-mapping-tools.org). The HeFESTo program is available at https://github.com/stixrude/HeFESToRepository. The Extended Burgers model is available at http://web.mit.edu/hufaul/www/Anelasticity.html.
