# Supplementary material for: Mantle heterogeneity caused by trapped water in the Southwest Basin of the South China Sea
Source: Nat Commun. 2023 May 11;14:2710. doi: 10.1038/s41467-023-38385-w (PMC10175291; doi:10.1038/s41467-023-38385-w)
Supplement: Supplementary file 1 — Supplementary Information [file 41467_2023_38385_MOESM1_ESM.pdf]

Supplementary Information for

Mantle heterogeneity caused by trapped water in the Southwest Sub-basin of the South China Sea

Jinyu Tian[1], Zhitu Ma[2], Jian Lin[1,3*], Min Xu[3], Xun Yu[2], Ba Manh Le[1], Xubo Zhang[3], Fan Zhang[3] & Laiyin Guo[1]

[1] Department of Ocean Science and Engineering, Southern University of Science and Technology, Shenzhen 518055, China

[2] State Key Laboratory of Marine Geology, School of Ocean and Earth Science, Tongji University, Shanghai 200092, China

[3] Key Laboratory of Ocean and Marginal Sea Geology, South China Sea Institute of Oceanology, Chinese Academy of Science, Guangzhou 510301, China

*Corresponding author: jianlin@scsio.ac.cn

Supplementary figures

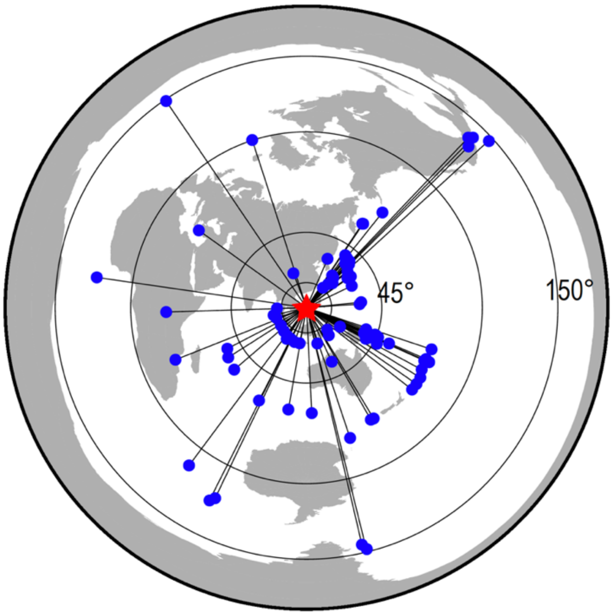

Supplementary Figure 1. Earthquakes used in this study. Blue dots represent the location of earthquakes determined by the IRIS seismic network. Red star is the location of the OBS array deployed in the study area.

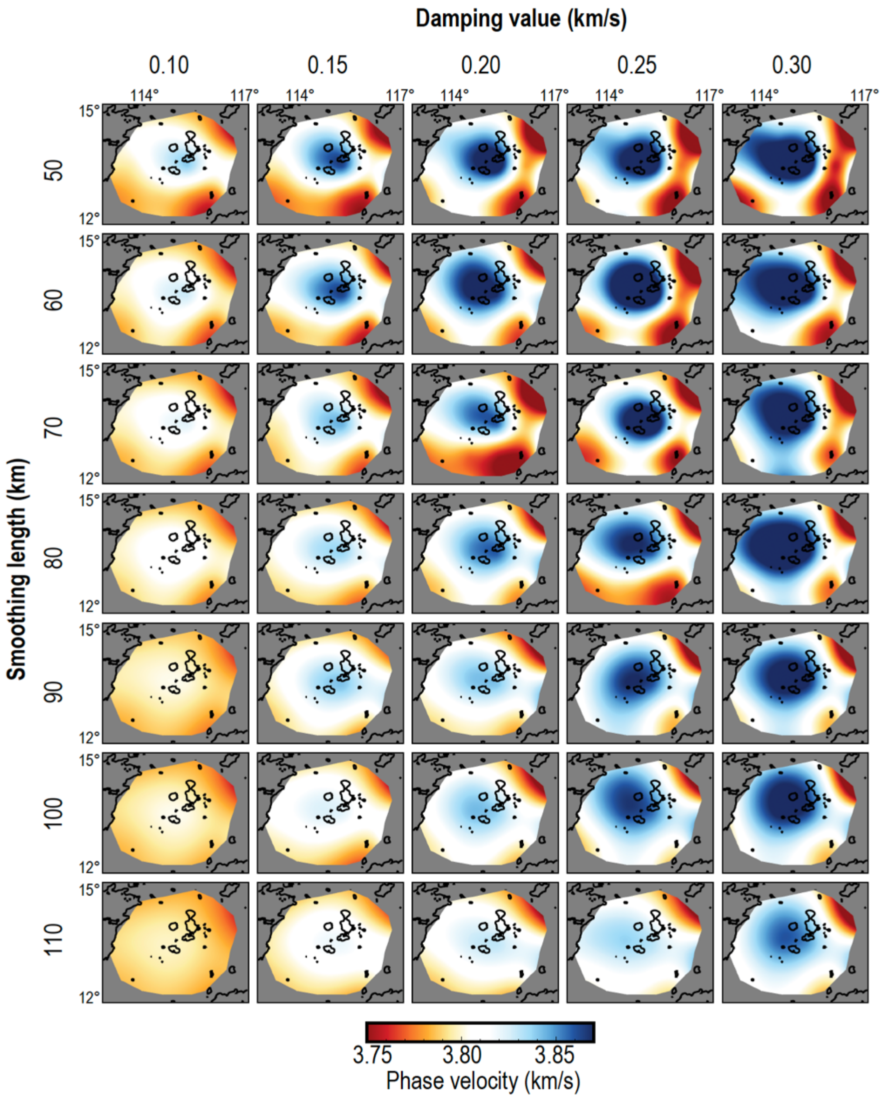

Supplementary Figure 2. Two-plane-wave tomography inversion results at 25 s as a function of the investigated parameters. The investigated damping value varies from 0.10–0.30 km s^{-1}, and the smoothing length varies from 50–110 km. In this study, values of 80 km and 0.2 km s^{-1} for the smoothing length and velocity damping parameters were chosen, respectively.

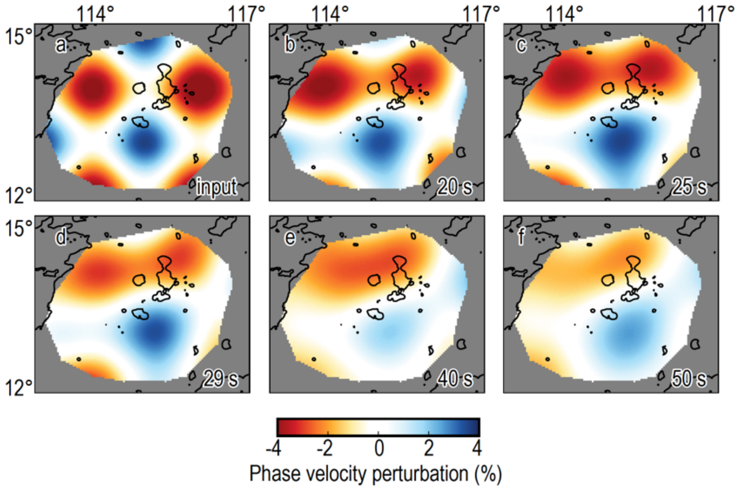

Supplementary Figure 3. Checkerboard resolution tests of the phase velocity models for multiple periods. a The sinusoidal checkerboard input model with the maximum velocity anomaly being ±4% related to 4 km s^{-1} in strength at the scale of 1°. Output inversion models at **b** 20, **c** 25, **d** 29, **e** 40, and **f** 50 s.

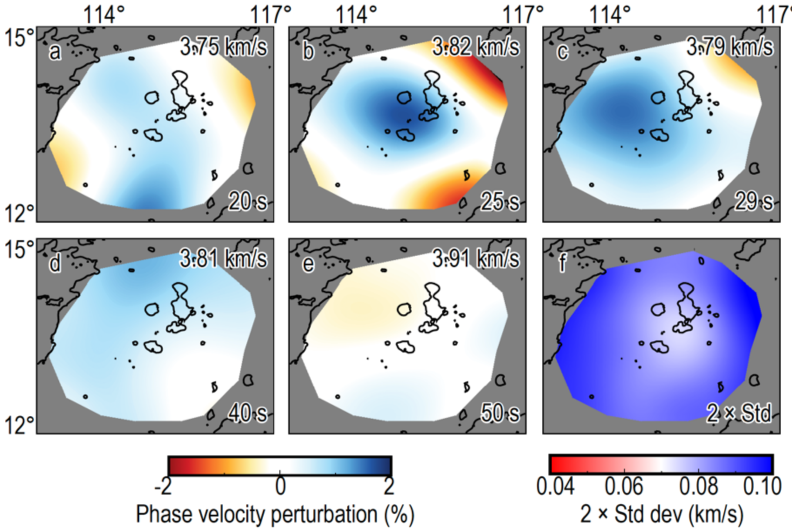

Supplementary Figure 4. Map of phase velocity and 2× standard errors. a–e The perturbation at each period is calculated by the average phase velocity, which is shown in the panels' top right. **f** The distribution of 2× standard errors of the phase velocities at 25 s.

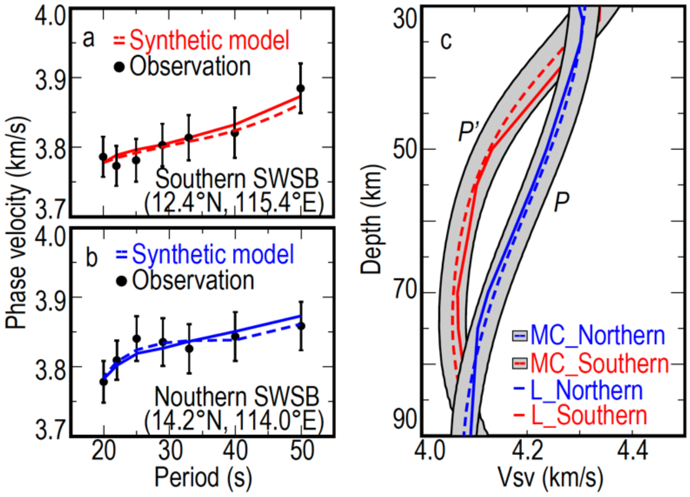

Supplementary Figure 5. Robustness test of the shear-velocity reduction. The black dots with vertical bars are the observed phase velocity models. The red dashed and solid lines represent the synthetic phase velocity and the inverted vertically polarized S-waves velocity (Vsv) models at the southern SWSB (12.4°N, 115.4°E) by the linearized method and the Bayesian Monte Carlo method, respectively. The blue dashed and solid lines represent those at the northern SWSB (14.2°N, 114.0°E). SWSB: Southwest Sub-basin.

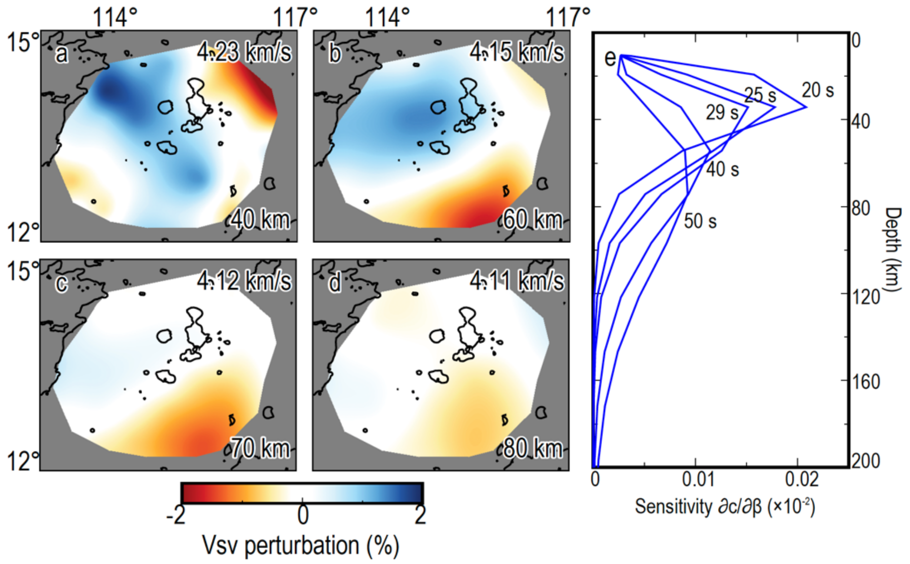

Supplementary Figure 6. Map of vertically polarized S-waves velocity models (Vsv) and sensitivity kernels. a–d The perturbation at each period is calculated by the average Vsv, which is shown in the panels' top right. **e** Phase velocity sensitivity kernels to Vsv for periods of 20, 25, 29, 40, and 50 s.

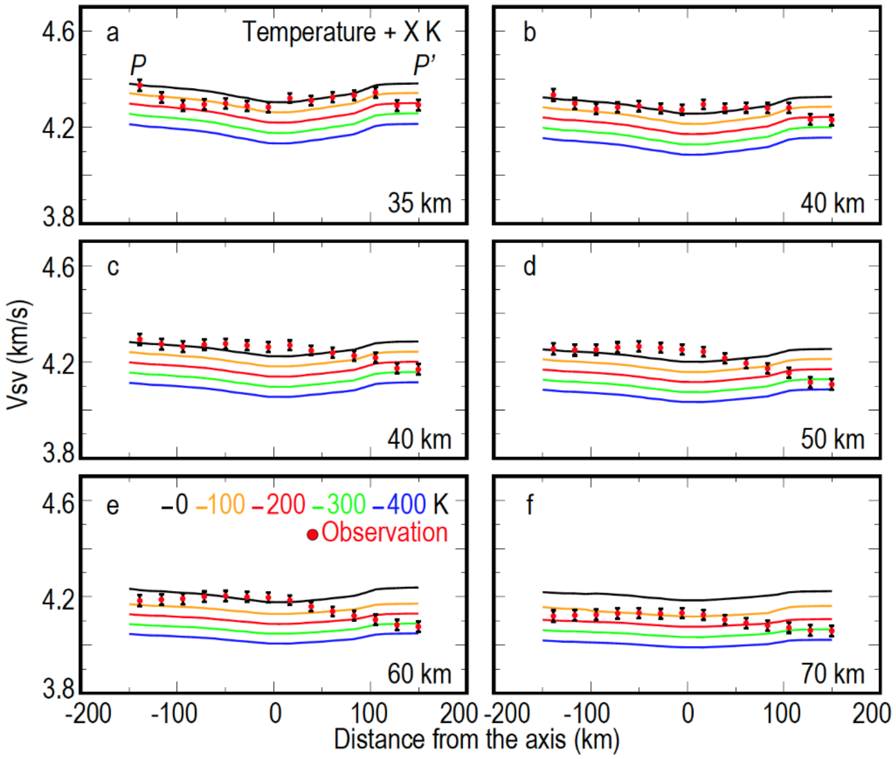

Supplementary Figure 7. Predicted vertically polarized S-waves velocity (Vsv) models by changing mantle temperature. The red dots with vertical black lines are the observed phase velocity models and estimated uncertainties. The black, gold, red, green, and blue lines represent the predicted Vsv by increasing the mantle temperature anomaly up 0, 100, 200, 300, and 400 K from 1600 K, respectively.

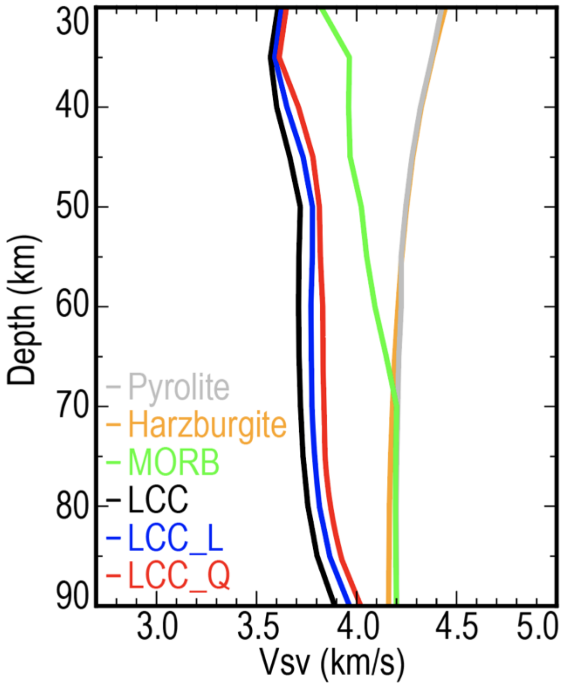

Supplementary Figure 8. Experimentally determined vertically polarized S-waves velocity (Vsv) models of different materials along the geotherm of *P'* (12.4°N, 115.4°E). Grey: pyrolite; Gold: harzburgite; Green: mid-ocean-ridge basalt (MORB); Black: average lower continental crust (LCC); Blue: LCC of Leizhou (LCC_L); Red: LCC of Qilin (LCC_Q).

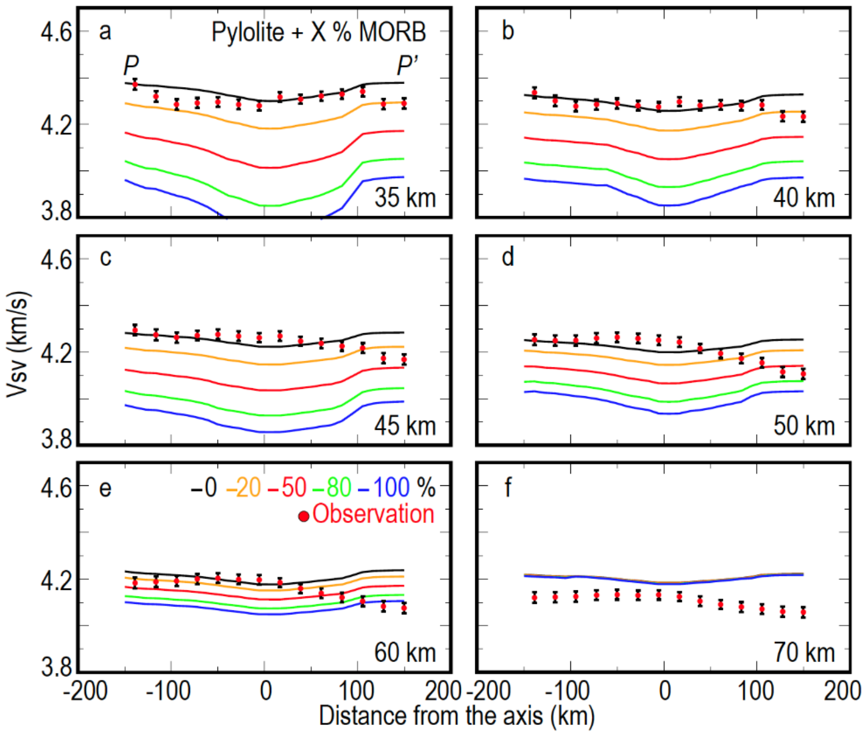

Supplementary Figure 9. Predicted vertically polarized S-waves velocity (Vsv) models for different proportions of mid-ocean-ridge basalt (MORB) mixed in pyrolite mantle. The red dots with vertical black bars are the observed phase velocity models and uncertainties. The black, gold, red, green, and blue lines represent the predicted Vsv by increasing the proportions of MORB in the pyrolite mantle from 0, 20%, 50%, 80%, and 100%, respectively.

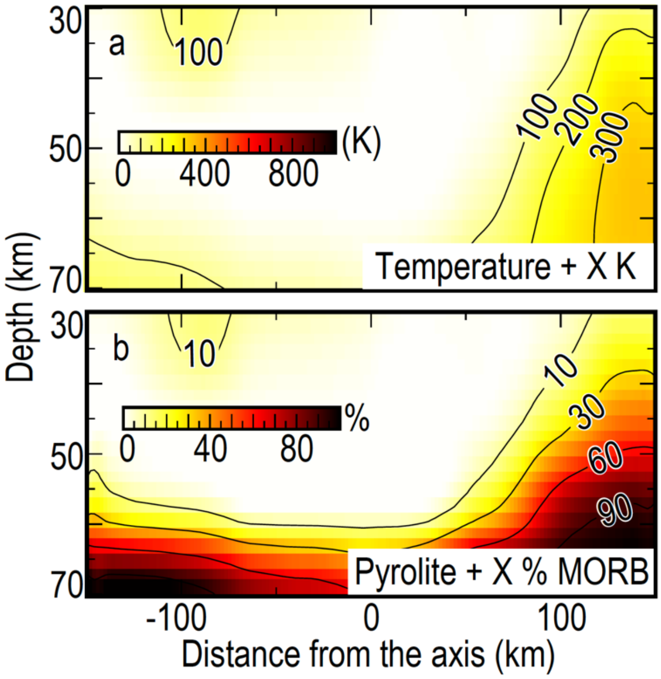

Supplementary Figure 10. Best fitting models of mantle composition variations along the cross-section P-P'. Fitting the observed Vsv structure by changing different mantle conditions: **a** mantle temperature. **b** adding the mid-ocean-ridge basalts (MORB) into the pyrolite mantle.

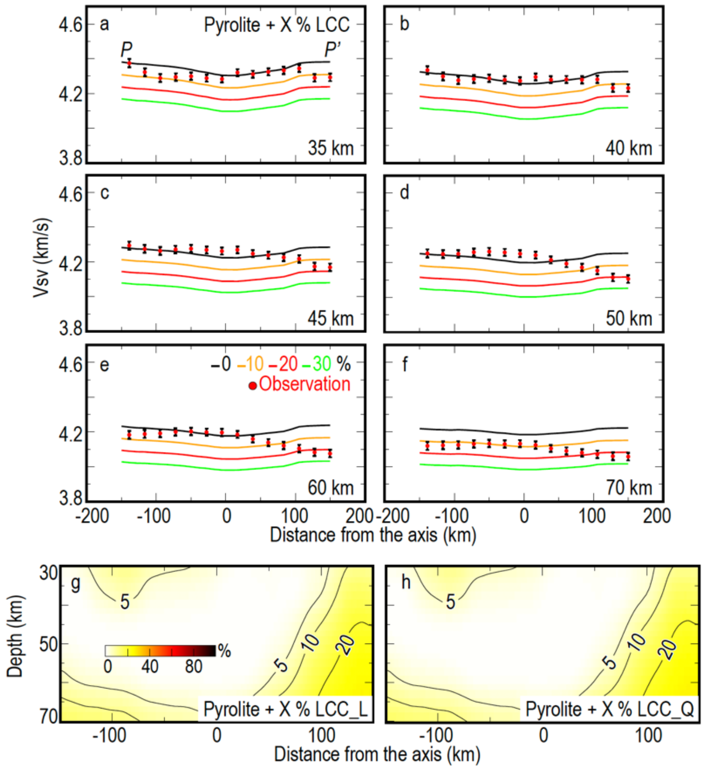

Supplementary Figure 11. Predicted Vsv models with different proportions of lower continental crust (LCC) mixed in pyrolite mantle. a–f The red dots with vertical black lines are the observed phase velocity models. The black, gold, red, green, and blue lines represent the predicted Vsv by increasing the proportions of LCC in the pyrolite mantle from 0, 10%, 20%, and 30%, respectively. **g–h** Fitting the observed Vsv structure by adding the LCC of Leizhou (LCC_L) and LCC of Qilin (LCC_Q) into the pyrolite mantle, respectively.

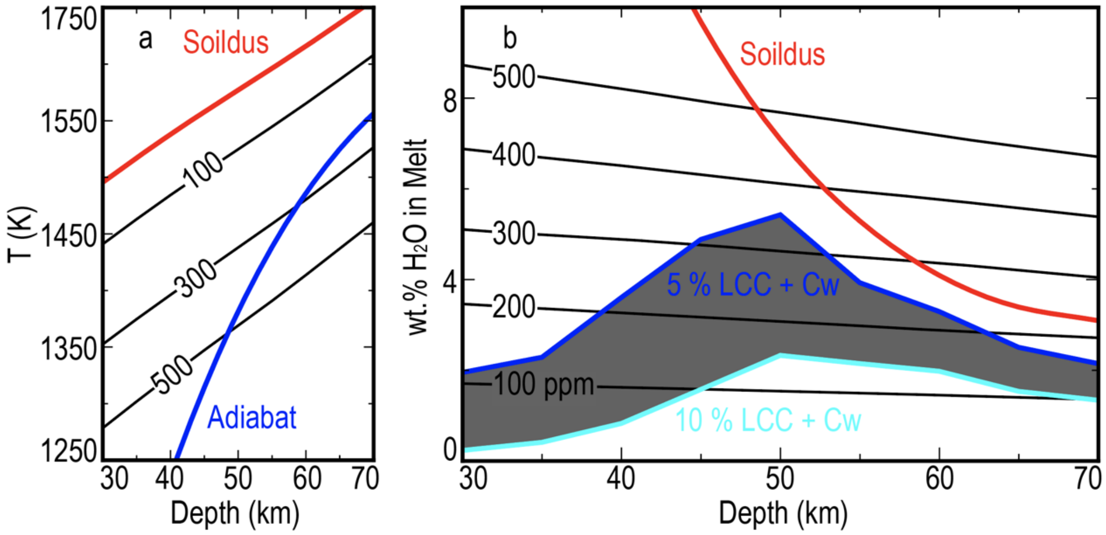

Supplementary Figure 12. Water content in the mantle required to stabilize a melt along the geotherm of *P’* (12.4°N, 115.4°E). a The red line represents the dry solidus of the pyrolite mantle. The blue line represents the adiabat line of point P’. Black lines represent the solidus of mantle with 100, 300, and 500 ppm of water. **b** The red line represents the water concentration necessary to stabilize a melt. Black lines represent the amount of water generated in near-solidus partial melts in the pyrolite mantle with 100–500 ppm water. Cyan and blue lines mark the estimated amount of water with 5% and 10% lower continental crust (LCC) in the mantle.

Supplementary Table 1

Bulk composition in mol%

Component	Pyrolite[1]	Basalt[1]	Harzburgite[2]	LCC[3]	LCC_L[4]	LCC_Q[4]
SiO_2	38.71	51.75	36.04	63.92	59.65	55.69
MgO	49.85	14.94	56.54	6.46	8.27	9.69
FeO	6.17	7.06	5.97	6.83	7.08	6.57
CaO	2.94	13.88	0.79	7.36	9.44	11.93
Al_2O_3	2.22	10.19	0.65	11.36	11.85	13.29
Na_2O	0.11	2.18	0.00	4.08	3.71	2.83

Supplementary References

1. Workman, R. K & Hart, S. R. Major and trace element composition of the depleted MORB mantle (DMM). *Earth Planet. Sci. Lett.* **231**, 53–72 (2005).
2. Xu, W., Lithgow-Bertelloni, C., Stixrude, L. & Ritsema, J. The effect of bulk composition and temperature on mantle seismic structure. *Earth Planet. Sci. Lett.* **275**, 70–79 (2008).
3. Hacker, B. R., Kelemen, P. B. & Behn, M. D. Continental lower crust. *Annu. Rev. Earth Planet. Sci.* **43**, 167–205 (2015).
4. Yu, J. H., Xu, X., O'Reilly, S. Y., Griffin, W. L. & Zhang, M. Granulite xenoliths from Cenozoic basalts in SE China provide geochemical fingerprints to distinguish lower crust terranes from the North and South China tectonic blocks. *Lithos* **67**, 77–102 (2003).
